# Gut immune cells—A novel therapeutical target for cardiovascular disease?

**DOI:** 10.3389/fcvm.2022.943214

**Published:** 2022-08-15

**Authors:** Naresh Ganesh, Emiel P. C. van der Vorst, Jens Spiesshöfer, Shun He, Mathias Burgmaier, Hannes Findeisen, Michael Lehrke, Filip K. Swirski, Nikolaus Marx, Florian Kahles

**Affiliations:** ^1^Department of Cardiology, Angiology and Intensive Care Medicine, University Hospital Aachen, Aachen, Germany; ^2^Interdisciplinary Center for Clinical Research (IZKF) and Institute for Molecular Cardiovascular Research (IMCAR), University Hospital Aachen, Aachen, Germany; ^3^Department of Pathology, Cardiovascular Research Institute Maastricht (CARIM), Maastricht University Medical Centre, Maastricht, Netherlands; ^4^Institute for Cardiovascular Prevention (IPEK), Ludwig-Maximilians-University Munich, Munich, Germany; ^5^Department of Pneumology and Intensive Care Medicine, University Hospital Aachen, Aachen, Germany; ^6^School of Medicine, Southern University of Science and Technology, Shenzhen, China; ^7^Department of Cardiology I—Coronary and Peripheral Vascular Disease, Heart Failure, University Hospital Münster, Münster, Germany; ^8^Icahn School of Medicine at Mount Sinai, Cardiovascular Research Institute, New York, NY, United States

**Keywords:** gut immune cells, intraepithelial lymphocytes, integrin β7, cardiovascular disease, myocardial infarction, atherosclerosis, heart failure, GLP-1

## Abstract

Despite scientific and clinical advances during the last 50 years cardiovascular disease continues to be the main cause of death worldwide. Especially patients with diabetes display a massive increased cardiovascular risk compared to patients without diabetes. Over the last two decades we have learned that cardiometabolic and cardiovascular diseases are driven by inflammation. Despite the fact that the gastrointestinal tract is one of the largest leukocyte reservoirs of our bodies, the relevance of gut immune cells for cardiovascular disease is largely unknown. First experimental evidence suggests an important relevance of immune cells in the intestinal tract for the development of metabolic and cardiovascular disease in mice. Mice specifically lacking gut immune cells are protected against obesity, diabetes, hypertension and atherosclerosis. Importantly antibody mediated inhibition of leukocyte homing into the gut showed similar protective metabolic and cardiovascular effects. Targeting gut immune cells might open novel therapeutic approaches for the treatment of cardiometabolic and cardiovascular diseases.

## Introduction

Scientific discoveries, advances in public health and innovations in medical care (including modern heart failure, lipid-lowering, and antiplatelet therapies) have contributed to a steady decline of mortality from cardiovascular disease in the last 50 years. Despite these improvements, cardiovascular diseases (such as coronary artery disease, myocardial infarction, or heart failure) continue to be the main cause of death worldwide (1, 2). Especially patients with diabetes are more vulnerable compared to those without diabetes and face a very high risk to die from cardiovascular disease (3). Recent evidence showed that sodium glucose linked transporter 2 (SGLT-2) inhibitors (4) and glucagon-like peptide 1 (GLP-1) receptor agonists (5) improved cardiovascular prognosis of patients with diabetes and high cardiovascular risk. Importantly, SGLT-2 inhibitors reduced heart failure endpoints in patients with or without diabetes (6, 7). Thus, SGLT-2 inhibitors emerged as first-line heart failure therapy in current guidelines (8). Despite these recent advances in heart failure and diabetes therapy there is still an urgent unmet clinical need to further reduce cardiovascular morbidity and mortality. Novel therapeutic approaches especially targeting atherosclerosis development and plaque stability must be identified and translated into promising drug targets. Targeting the immune system might be a novel therapeutical approach to protect against cardiovascular disease and its life-threatening complications.

## The role of inflammation in cardiovascular disease

Data from the last two decades have shown that leukocytes and inflammatory processes are important players during the development of cardiovascular diseases such as atherosclerosis, myocardial infarction, and heart failure (9–11). Elegant experimental studies have identified inflammatory organ cross talk networks between the heart, the bone marrow, the spleen, and the sympathetic nervous system which could explain how immune cells control and accelerate the development and progression of cardiovascular disease. These observations suggest that after myocardial infarction enhanced sympathetic nervous activity releases noradrenalin in the bone marrow niche, which results in increased hematopoietic stem cell (HSC) activity and emigration to extramedullary sites. Increased production of myeloid immune cells (myelopoiesis) in the bone marrow results in higher numbers of circulating myeloid immune cells that are recruited to atherosclerotic plaques in higher numbers, accelerating plaque growth and inflammation and decreasing plaque stability thus increasing the risk of re-infarction (12–14). This feedback loop and vicious cycle might be one of the explanations why patients after a myocardial infarction are at increased risk to experience re-infarction.

Immune cells play a crucial role in atherosclerosis formation and subsequent plaque growth. Following intimal lipid accumulation and endothelial dysfunction monocytes migrate into the vessel wall [reviewed in Swirski and Nahrendorf (12)]. Newly-infiltrated monocytes differentiate into macrophages which recognize and ingest lipids that have accumulated in the intima as a consequence of hypercholesterolemia. After lipid ingestion macrophages become lipid- rich “foam cells,” thus activating various inflammatory pathways leading to activation of other immune (including T and B lymphocytes, neutrophils, and dendritic cells) and non-immune cells [including endothelial cells, platelets, smooth muscle cells; reviewed in Swirski and Nahrendorf (12)].

In line with these findings circulating leukocyte numbers and biomarkers of inflammation such as high-sensitivity C-reactive protein and interleukin-6 are associated with an increased risk of cardiovascular events in humans (15–17). In order to prove the inflammatory hypothesis of atherothrombosis several large cardiovascular outcome trials validating drugs which target inflammatory pathways have been conducted. In the Canakinumab Anti-Inflammatory Thrombosis Outcomes Study (CANTOS) canakinumab, a therapeutic monoclonal antibody targeting interleukin-1β, showed a 15% risk reduction of non-fatal myocardial infarction (MI), non-fatal stroke, or cardiovascular mortality in patients with previous myocardial infarction and hs-CRP above 2 mg/L (18). In this trial canakinumab was associated with a higher incidence of fatal infection compared to placebo. While this study for the first time demonstrated that an anti-inflammatory drug is able to improve cardiovascular outcomes, the Food and Drug Administration (FDA) rejected clinical approval of canakinumab for cardiovascular disease most likely due to safety concerns and only modest outcome effects. Colchicine, an NACHT, LRR, and PYD domains-containing protein 3 (NLRP3)-inflammasome inhibitor, was investigated in patients with a recent myocardial infarction [Colchicine Cardiovascular Outcomes Trial—COLCOT (19)] and in patients with chronic coronary disease [In the Low-Dose Colchicine 2—LoDoCo2 trial (20)]. In both studies low-dose colchicine significantly reduced cardiovascular events. However, the incidence of death from non-cardiovascular causes was numerically higher in the colchicine group than in the placebo group which requires further investigations. While these studies proved the inflammatory hypothesis of cardiovascular disease, interleukin-1β (canakinumab) or the NLRP3-inflammasome (colchicine) might not be the optimal drug candidates for this purpose. Thus, alternative therapeutical targets modulating inflammation need to be identified to improve prognosis in patients with cardiovascular disease.

## Gut immune cells and cardiovascular disease

The gut microbiome and intestinal barrier dysfunction (“leaky gut”) have emerged as potential contributors to the development of cardiovascular disease and are currently intensively studied (21–23). Despite the fact that the gastrointestinal tract (GI tract) is one of the largest leukocyte reservoirs of our bodies containing very high numbers of T lymphocytes, plasma cells, eosinophils, and macrophages (24–26), the relevance of gut immune cells for cardiovascular disease is largely unknown. Integrin β7 (*Itgb7*) is expressed on circulating leukocytes and mediates immune cell migration selectively into the gut without affecting relevant chemotaxis into other tissues (27–31). Thus, *Itgb*7^−/−^ (hereafter β7^−/−^ mice) display a useful tool to study selective gut immune cell deficiency, since these mice show strongly reduced leukocyte numbers (especially intraepithelial lymphocytes: αβ and γδ T cells, B cells, and myeloid cells) in the gut. A recently published study investigated whether gut immune cells play a role in atherosclerosis development in mice (32). β*7* deficient mice on *Ldlr*^−/−^ background fed a high cholesterol diet (HCD) showed no difference in weight gain, but had lower levels of plasma total cholesterol, a reduction in circulating Ly-6C^high^ and Ly-6C^low^ monocytes and smaller aortic lesions with an ~50% reduction in plaque size and volume (32). To elucidate the question whether gut immune cells can be therapeutically targeted for cardiovascular disease the authors injected anti-integrin β7 antibodies (which block immune cell homing to the gut) into *Ldlr*^−/−^ mice and found that these mice had attenuated atherosclerosis (32). These findings show that gut immune cells accelerate lesion growth while antibody mediated integrin β7 neutralization protects against atherosclerosis formation in mice.

Patients with diabetes and obesity show more severe atherosclerosis and higher cardiovascular risk than those without diabetes. Based on the promising findings on targeting intestinal leukocytes to treat atherosclerosis it is important to investigate the functional relevance of gut immune cells in cardiometabolic diseases. Several studies in obese mice and men demonstrated major changes to the intestinal immune cell landscape in the obese state compared with the lean state. During metabolic disease the gut contains increased numbers of γδ T cells, macrophages, dendritic cells, NK cells, CD8+ T cells (αβ TCR), and Th1 T cells and a reduction in Treg T cells, anti-inflammatory IgA+ antibody-secreting cells (ASCs) and eosinophils. These changes in immune compartments are associated with an inflammatory environment that is linked with intestinal barrier dysfunction, intestinal dysbiosis and a loss in bacterial diversity during diet-induced obesity (33–40). Interestingly, mice lacking gut immune cells (β7^−/−^, Integrin alpha E knockout—*Itgae*^−/−^ or C-C chemokine receptor type 9 knockout—*Ccr*9^−/−^ mice) fed a diet high in fat, sodium and sugar showed improved glucose tolerance, less weight gain, and reduced white adipose tissue inflammation (reduced numbers of Ly-6C^high^ monocytes, neutrophils, and macrophages) (32, 35).

**Figure 1:**
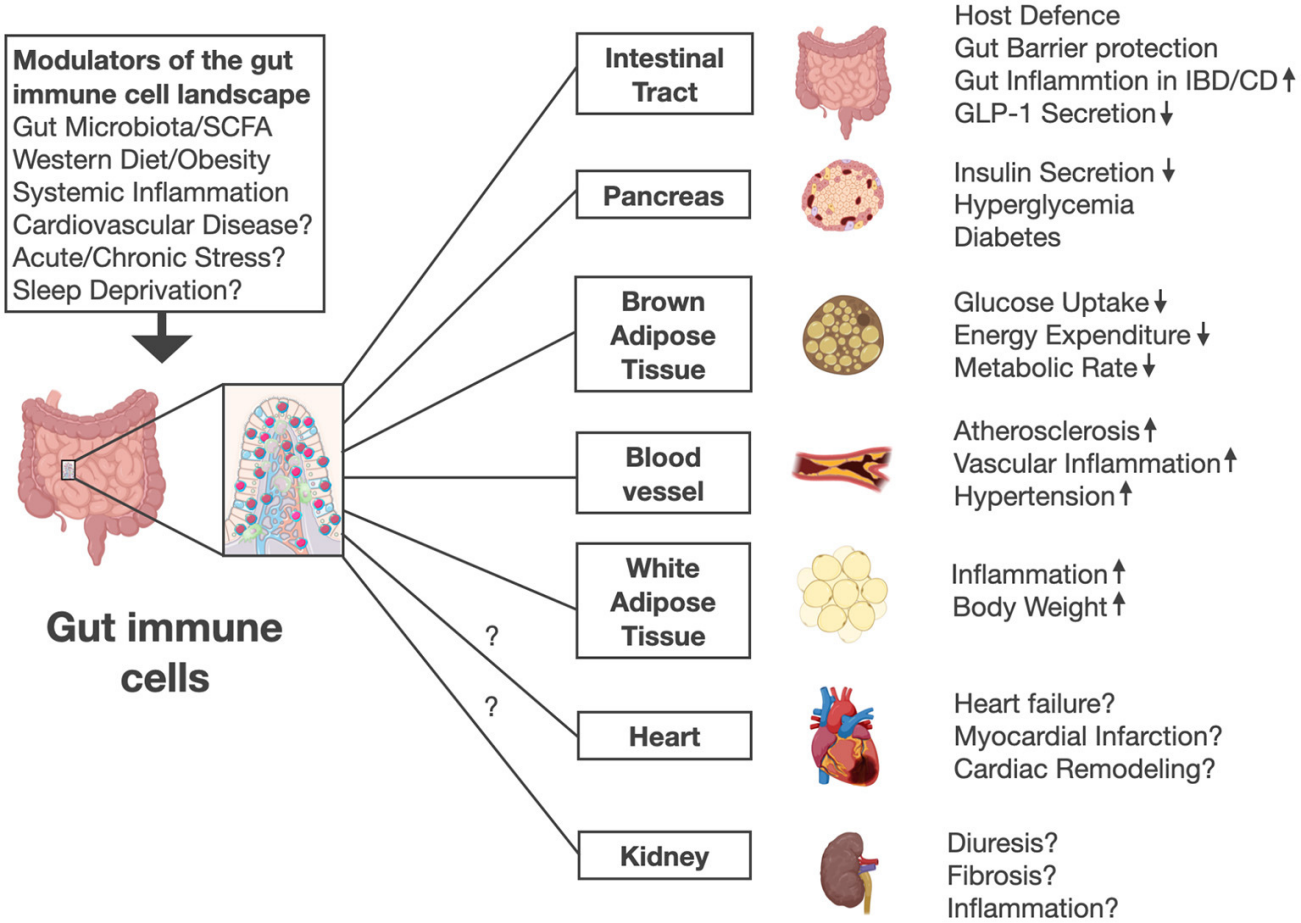
Gut immune cells are modulated by gut microbiota, short chain fatty acids (SCFA), diet and systemic inflammation in mice. Future work is needed to investigate whether other stimuli and lifestyle factors including stress or sleep disorders affect gut immune cell numbers and activation. Intestinal immune cells mediate pleiotropic effects in various organs beyond their role in host defense and gut barrier protection. Mice lacking gut immune cells show increased insulin secretion, improved glucose control, higher energy expenditure, lower body weight, less high fat diet-induced adipose tissue inflammation, and increased circulating GLP-1 levels. Thus, gut immune cell deficient mice were protected against diabetes, obesity, hypertension and atherosclerosis. Future work needs to address whether gut immune cells might affect other diseases including heart failure, myocardial infarction, and kidney function. The illustration was modified from Biorender (https://Biorender.com).

In contrast to wild-type control mice, β7^−/−^ mice were also protected from hypertension (a key feature of the metabolic syndrome), indicating that gut immune cells aggravate adverse cardiometabolic consequences of high-fat diet (32). Mechanistically β7^−/−^ mice on chow diet showed improved glucose tolerance by increased insulin secretion, expended more energy, produced more heat and had lower levels of fasting triglycerides without differences in hepatic secretion of triglycerides or fat absorption (32). These results suggest metabolic rate to be increased in the absence of gut immune cells. While diet-induced obesity is linked with intestinal barrier dysfunction and a loss in bacterial diversity (33–40), the beneficial metabolic effects in β7^−/−^ mice were resistant to antibiotic treatment and not associated with gut permeability abnormalities (32). These findings support a direct effect of gut immune cells on metabolic and cardiovascular disease independent of gut barrier integrity or microbiome changes (Figure 1).

## GLP-1: Mechanistic link connecting gut immune cells and cardiovascular disease?

**Figure 2:**
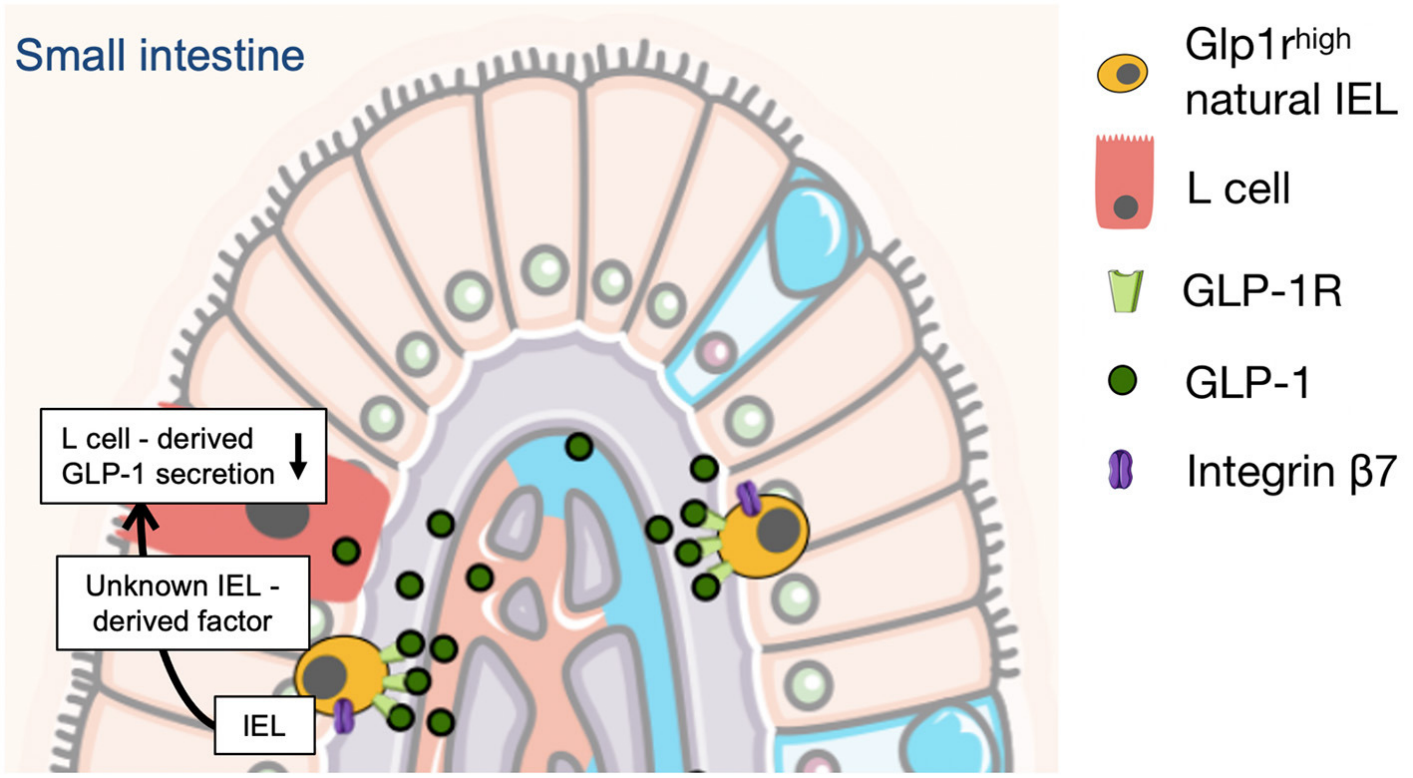
L-cell derived GLP-1 secretion is controlled and limited by gut intraepithelial (IEL) lymphocytes in mice. This is mediated through a negative feedback mechanism in which gut IEL GLP-1 receptor activation directly inhibits L cell derived GLP-1 secretion through a still unknown IEL-derived mediator (32). The illustration was modified from Servier Medical Art (https://smart.servier.com/).

In response to food intake the gut incretin hormone GLP-1 (glucagon-like peptide-1) is secreted by intestinal L cells leading to insulin secretion and glucose control (41). Pharmacological activation of the GLP-1 receptor is currently used for the treatment of patients with type 2 diabetes (42). Beyond their glucoregulatory function GLP-1 receptor agonists exert pleiotropic protective cardiovascular effects in different organ systems (43) and improved cardiovascular outcomes in diabetic patients at high cardiovascular risk (44–49). Besides food intake GLP-1 secretion is induced by acute inflammatory stimuli including LPS, IL-6, and IL1b. This was found to be mediated by IL-6 signaling in L-cells (50, 51). Consistently mice and patients with acute [sepsis (51) or myocardial infarction (52)] or chronic inflammatory cardiovascular diseases [coronary artery disease (53) or heart failure (54)] show increased circulating GLP-1 levels independent of food intake. Elevated GLP-1 levels were independently associated with mortality in patients with sepsis or myocardial infarction (55, 56). Mechanistic experimental studies suggest upregulation of GLP-1 secretion to be an endogenous protective counter-regulatory response circuit (52). Interestingly gut immune cell deficient mice (β7^−/−^) which are protected from atherosclerosis, hypertension, obesity and diabetes have higher plasma GLP-1 levels (32, 35). Since gut immune cells (in particular intraepithelial αβ and γδ T lymphocytes) express high levels of the GLP-1 receptor (32, 57) it was elucidated whether these cells directly regulate GLP-1 secretion from intestinal L cells to modulate cardiometabolic disease. For this purpose the authors of a previous study generated mixed bone marrow chimeras (bmGlp1r^−/−^β7^−/−^) with selective GLP-1 receptor deficiency in β7^+^ gut immune cells and unaffected GLP-1 receptor expression on all other cell types. Mice with selective gut immune cell GLP-1 receptor deficiency showed higher plasma levels of GLP-1, were more glucose tolerant, presented with less hypercholesterolemia and developed smaller atherosclerotic plaques with fewer aortic leukocytes (32). Further mechanistic experiments identified a negative feedback mechanism in which gut immune cell GLP-1 receptor activation was found to directly inhibit L cell derived GLP-1 secretion through a still unknown gut immune cell derived mediator (32). These findings suggest that GLP-1 receptor deficiency in gut immune cells limits development of cardiovascular and cardiometabolic disease through upregulation of systemic GLP-1 availability (Figure 2). In other words GLP-1 might be one of the underlying mechanistic links connecting gut immune cells and cardiovascular disease.

## Conclusion and future perspectives

Cardiometabolic diseases are characterized and driven by systemic inflammation. Recent data indicate that in cardiovascular and metabolic diseases inflammation is not limited to the vessel wall, bone marrow, spleen or adipose tissue. First experimental evidence suggests an important relevance of immune cells in the intestinal tract for the development of metabolic and cardiovascular disease in mice. Mice specifically lacking gut immune cells are protected against obesity, diabetes, hypertension and atherosclerosis. Importantly anti-Integrin-β7 antibody mediated inhibition of leukocyte homing into the gut showed similar protective metabolic and cardiovascular effects as genetic models. Based on these observations targeting gut immune cells and in particular Integrin-β7 might open novel therapeutic approaches for the treatment of cardiometabolic and cardiovascular disease. Therapeutic targeting of gut immune cell trafficking is well-established and safe in patients suffering from inflammatory bowel diseases (IBDs), including Crohn's disease (CD), and ulcerative colitis (UC). Blocking intestinal immune cell homing by Vedolizumab, an anti-α4β7 antibody, has become an important pillar of IBD therapy (58–61). Therefore, it is tempting to speculate that antibody-mediated blockage of intestinal leukocyte homing might be a promising drug target for patients with cardiovascular disease. Especially the recent identification of a previously unrecognized crosstalk between gut immune cells and the incretin system (increased GLP-1 secretion in the absence of gut immune cells) might open new therapeutic avenues for vulnerable high-risk patients with diabetes and cardiovascular disease. However, several key questions remain: Can we translate the experimental observations on gut immune cells and cardiovascular and cardiometabolic effects to patients? Is intestinal immune cell trafficking and activation relevant for human cardiovascular disease? How exactly do gut leukocytes accelerate atherosclerosis formation, hypertension, and diabetes? Is GLP-1 the only mechanistic link between gut immune cells and cardiovascular actions or do other incretin hormones such as GIP (glucose-dependent insulinotropic peptide) or GLP-2 or any other as of yet unknown factors play a role? Does gut immune cell trafficking or activation affect plaque inflammation by increasing circulating monocytes and neutrophils (due to upregulated hematopoiesis)? Do gut immune cells control local macrophage proliferation in atherosclerotic lesions? Beyond their role in atherogenesis what is the effect of blocking intestinal leukocyte homing on other manifestations of cardiovascular disease like cardiac remodeling after myocardial infarction or heart failure? What is the effect of cardiovascular events (i.e., myocardial infarction, stroke, or decompensated heart failure) on intestinal immune cell homing and activation? Are gut immune cell numbers and or activation status associated with cardiovascular risk in patients?

Answering these questions may be essential to better understand how intestinal leukocytes control development of cardiometabolic and cardiovascular disease and to evaluate whether the above mentioned promising experimental observations could the translated into clinical practice. Targeting gut immune cells might be a future therapeutical approach to prevent and suppress the devastating consequences of cardiovascular disease.

## Author contributions

FK and NG drafted the manuscript, which was critically revised by all authors. All authors provided intellectual input and gave final approval.

## Funding

This study was supported by grants from the European Research Area Network on Cardiovascular Diseases (ERA-CVD and BMBF, Grant No. JTC-2019, MyPenPath-01KL2004) and from The European Foundation for the Study of Diabetes (EFSD)/Novo Nordisk Foundation (NNF2OSA0066111) to FK and grants from the Deutsche Forschungsgemeinschaft (SFB TRR 219 M-03) and Interreg V-A grant EURlipids to ML. NM was supported by grants from the Deutsche Forschungsgemeinschaft (German Research Foundation; TRR 219; Project-ID 322900939 [M03, M05]) as well as the CORONA Stiftung, Germany. EV was supported by a grant from the Interdisciplinary Center for Clinical Research within the faculty of Medicine at the RWTH Aachen University and NWO-ZonMw Veni (91619053).

## Conflict of interest

Author FK served as a consultant to Bayer and Novo Nordisk and served as a speaker for Novo Nordisk. Author NM has received support for clinical trial leadership from Boehringer Ingelheim, Novo Nordisk, served as a consultant to Bayer, Boehringer Ingelheim, Merck, Novo Nordisk, AstraZeneca, BMS, received grant support from Boehringer Ingelheim, Merck, Novo Nordisk, and served as a speaker for Bayer, Boehringer Ingelheim, Merck, Novo Nordisk, Lilly, BMS, and AstraZeneca. Author ML received grants and personal fees from Boehringer Ingelheim, MSD, Novo Nordisk and personal fees from Amgen, Sanofi, Astra Zeneca, Bayer, Lilly, Daiichi Sankyo, Novarits, Amarin. The remaining authors declare that the research was conducted in the absence of any commercial or financial relationships that could be construed as a potential conflict of interest.

## Publisher's note

All claims expressed in this article are solely those of the authors and do not necessarily represent those of their affiliated organizations, or those of the publisher, the editors and the reviewers. Any product that may be evaluated in this article, or claim that may be made by its manufacturer, is not guaranteed or endorsed by the publisher.
